# Frailty modulates the predictive value of performance status in older adults living with cancer

**DOI:** 10.1007/s40520-025-03203-4

**Published:** 2025-10-30

**Authors:** Daniela Patino-Hernandez, Mario Ulises Pérez-Zepeda, Natalia Sánchez-Garrido, Alejandro Eliú Cedillo, Eduardo Cárdenas-Cárdenas

**Affiliations:** 1 Oncology Unit, Internal Medicine Department, Hospital Universitario San Ignacio, Bogotá, Colombia; 2 Semillero de Neurociencias y Envejecimiento, Aging Institute, Medical School, Pontificia Universidad Javeriana, Bogotá, Colombia; 3 Research Division, Instituto Nacional de Geriatría, Mexico City, Mexico; 4 Facultad de Psicología, Universidad Anáhuac México, Huixquilucan Edo. de México, Mexico City, Mexico; 5 Coordinación de Enseñanza, Instituto de Seguridad Social y Salud de los Trabajadores del Estado, Centro Médico Nacional 20 de Noviembre, Mexico City, Mexico

**Keywords:** Days in bed, Performance status, Cancer, Older adults, Frailty.

## Abstract

**Background:**

Cancer prognosis in older adults is complicated by age-related vulnerabilities such as frailty, which may impact the performance of traditional prognostic tools, which may not accurately reflect risk in this population.

**Aims:**

This study aimed to assess whether frailty modifies the association between a modified performance status (PS) —constructed from days spent in bed, physical activity, and disability—and mortality risk in older adults with cancer.

**Methods:**

We conducted a secondary analysis of the Mexican Health and Aging Study (MHAS), including individuals ≥50 years with incident cancer and active treatment, with follow-up through 2021. A frailty index (FI) was calculated using a 33-item deficit accumulation model. Modified PS was constructed using data on days in bed, functional limitations, and physical activity. Participants were stratified into low (<0.25) and high (≥0.25) frailty levels. Cox regression and Kaplan–Meier curves were used to assess associations with all-cause mortality.

**Results:**

Among 318 participants (mean age 68.0 years; 62.6% women), frailty and performance status were both associated with mortality. In individuals with low frailty, modified PS categories 2 and 4 were significantly associated with higher mortality (HR 5.46 [95% CI: 1.24–24.04], and HR 8.01 [1.50–42.66], respectively). Among those with high frailty, only age remained a significant predictor of mortality (HR 1.04 [1.00–1.07]).

**Discussion:**

Frailty significantly modifies the prognostic value of performance status. In frail older adults with cancer, the predictive utility of performance status tools is reduced.

**Conclusions.:**

Incorporating frailty assessment may enhance risk stratification and clinical decision-making in older adults with cancer.

## Highlights

Frailty alters the predictive value of performance status in older cancer patients.

Higher frailty levels reduce the prognostic power of physical performance measures.

Social vulnerability strongly influences mortality, independent of frailty status.

Geriatric assessments are crucial for improving cancer prognosis in older adults.

## Introduction

Cancer treatments are not a one-size-fits-all approach, and even when age is a well-known risk factor for developing malignant neoplasms, course and treatment options are scarcely studied in aging individuals, leading in many cases to worse outcomes, due to increased vulnerability of these individuals [1]. Frequently, impaired functional and cognitive status increase the risk for treatment-related toxicity, leading to a decline of overall health or even death [2]. For example, social vulnerability; understood as the degree to which susceptibility to health problems may be modulated by the complexity of a particular social situations (e.g., family members, friends, community engagement, financial income, etc.), has shown to be a relevant factor into health outcomes of older adults [3, 4]. Moreover, socioeconomic and cultural factors have important roles in these outcomes of older adults suffering from cancer [5].

On the other hand, frailty — defined as an age-related increase in susceptibility to common stressors— is associated with multiple adverse outcomes across common conditions of the older adults [6, 7]. Evidence has shown that this condition can shape the prognosis of individuals (e.g., diseases, treatments, emotional crises), particularly those diagnosed with cancer [8, 9]; challenging the results of many tools adopted to predict adverse outcomes in younger individuals.

Regarding cancer prognosis, functional status has been evidenced as having a better prognostic value in oncology than other parameters –such as age– accurately establishing prognosis, tolerance, quality of life and treatment response [10, 11]. Indeed, two of the most used instruments for assessing performance status in oncological practice include the Eastern Cooperative Oncology Group (ECOG) and the Karnofsky scale [12], both of which include functional performance assessment. However, these instruments have been criticized for being highly subjective and not being able to detect real-time changes of potential clinical relevance (e.g., low prognostic value for older adults) [11, 13]. Additionally, these evaluations were not initially intended to assess older adults and might not have the same clinimetric properties as in younger individuals [14], leading to undertreatment if functional status were to be underestimated.

Therefore, the objective of this study is to determine whether frailty modulates the association between a composite performance status indicator—based on days in bed, physical activity, and functional disability—and all-cause mortality in older adults with cancer. We hypothesize that frailty attenuates the predictive value of performance status, with limited prognostic utility in individuals with high frailty. This work seeks to refine prognostic stratification in geriatric oncology and promote the integration of frailty assessment into clinical care.

## Methods

### Design and sample

This is a secondary analysis of the Mexican Health and Aging Study (MHAS), a cohort with a national-level representative sample from Mexican individuals aged 50-years or older. In brief, this study aims to determine the factors that impact aging in Mexican individuals and consists of several waves, with a baseline assessment in 2001; follow-up data are available for the years 2003, 2012, 2015, 2018 and 2021; information from a wide array of topics was obtained from face‒to‒face interviews conducted by trained personnel. Further details about MHAS can be found elsewhere [15–17].

**Fig. 1:**
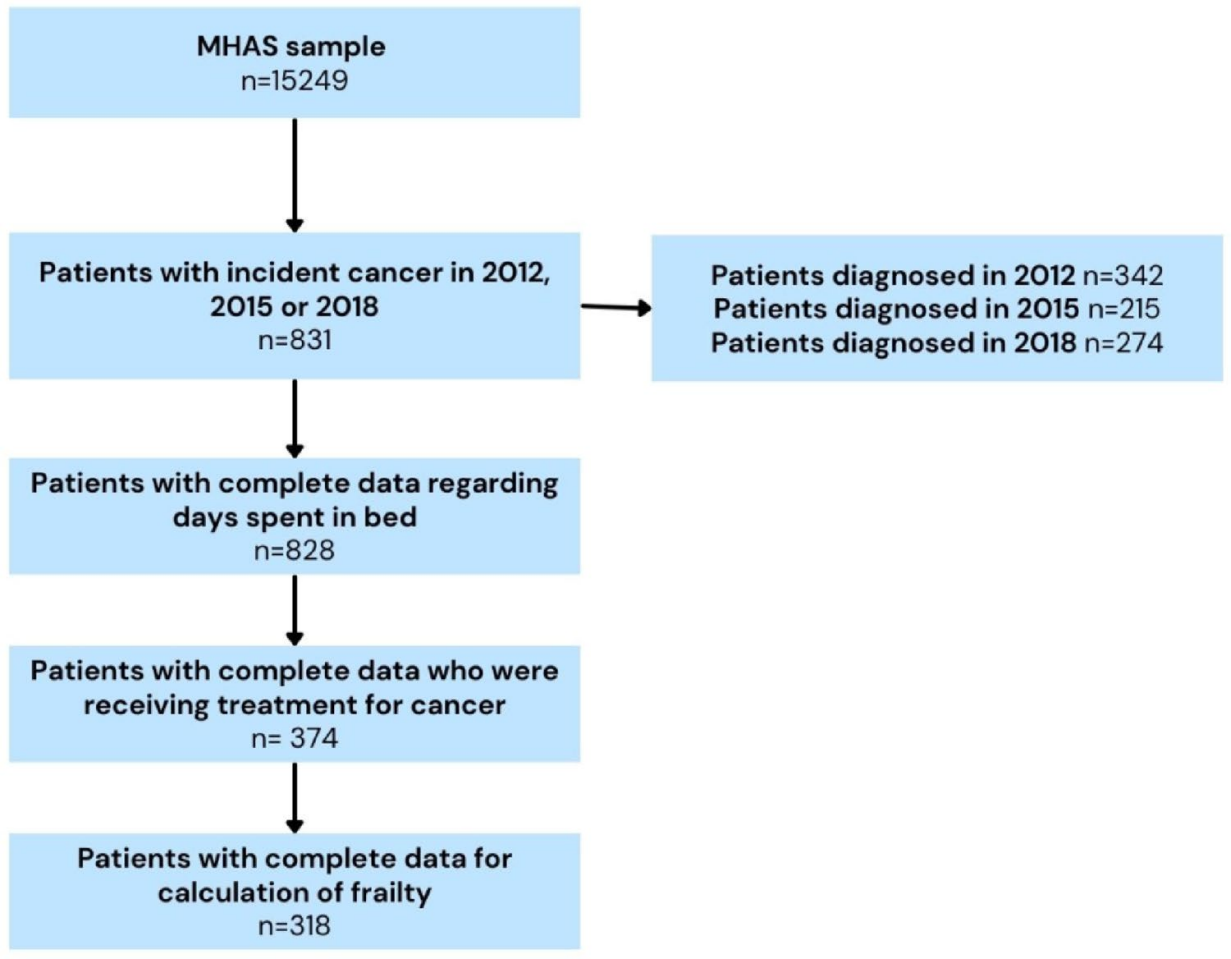
Sample flow chart

For this work, we used data from MHAS assessments from 2012, 2015 and 2018 and follow-up information on survival status through 2021. We extracted the baseline variables from the main questionnaires, and the next-of-kin (NOK) questionnaires were used for information regarding mortality. All individuals ≥ 50 years old who answered directly to the baseline interview and with complete data (both baseline and follow-up) were included (Fig. 1).

### Variables

#### Cancer status

Subjects who reported having cancer were identified by asking the following question: ‘Has a doctor or medical personnel ever diagnosed you with cancer?’ If the individual answered ‘yes’ a follow-up question regarding the type of cancer was asked (including breast, cervix, endometrial/uterine, liver, stomach, pancreas, prostate, colorectal, and lung) (see Fig. 1). Our sample was composed only of ‘incident’ cases, meaning that they had not previously been diagnosed with cancer and did not refer having cancer in previous waves. We further restricted our sample to include only subjects who reported being treated currently in any of the subsequent follow-up interviews. These two criteria (new cancer and currently receiving treatment) were introduced to maximize the approximation to ‘real-world’ and actual clinical practice. Finally, the type of cancer and treatment (surgery, radiotherapy or chemotherapy) were included for descriptive purposes.

#### Modified performance status

The Eastern Cooperative Oncology Group (ECOG) scale is based on the time spent in bed, with 0 representing full functionality for asymptomatic patients and 4 being bedridden [13]. Since MHAS does not include measurements to assess cancer prognosis due to its general scope of aging, we created a composite measure modeled after ECOG performance status, constructed from available MHAS variables related to days in bed, physical activity, and disability. This variable will be further referred to as “Modified Performance Status” throughout the text. Firstly, number of days spent in bed per year was obtained through the following question: ‘Owing to sickness or injury, during the last 12 months, how many days did you stay in bed for at least half the day?’ [18]. Secondly, physical activity was assessed by answering yes or no to the following question: ‘On average, during the previous two years, have you exercised or had done vigorous physical activity three times a week or more? ´ Finally, basic and instrumental activities of daily living were included, as follows: the MHAS includes a set of 19 of these activities (please see supplementary material); whenever an older adult answered that they were not able to perform an activity, a score of 1 was assigned; if no difficulty was present, the score was 0; and if help was required to perform the activity, a score of 0.5 was assigned. The scores were summed, resulting in a score of 0 (no difficulties in activities of daily living) or up to 19, the highest possible score (impossibility of performing all the assessed activities). More detail can be found in supplementary Table 1.

We then categorized the Modified performance status score into 5 categories: 0 (< 50% of days spent in bed during the previous year, no disability and reported physical activity), 1 (< 50% of days spent in bed during the previous year, no disability and no report of physical activity), 2 (< 50% of days spent in bed during the previous year, disability and no report of physical activity), 3 (> 50% of days spent in bed during the previous year, no disability and no report of physical activity), and 4 (> 50% of days spent in bed during the previous year, disability and no report of physical activity).

#### Mortality

All-cause mortality was our outcome of interest, including time to event, for survival analysis. For those who survived, follow-up days were calculated as the difference between the interview date in 2001 and that from 2021; in a similar fashion, time to event was calculated as the difference in days from the baseline interview to the reported date of death.

#### Frailty index

The frailty index (FI) was calculated from 33 variables related to self-reported health, comorbidities, depressive symptoms and other symptoms, based on the deficit accumulation approach [19]. This index has been previously validated in the MHAS dataset [20]. A modified version of the original 52-item FI was used, excluding variables incorporated into the performance status proxy to avoid collinearity. (see above). Characteristics of the index, such as: prevalence of the deficits, coding and definition from MHAS are available in supplementary Table 2. For analytical purposes, we categorized the FI into two levels: Low frailty if < 0.25 and High frailty if the FI was ≥ 0.25, according to prior studies using the MHAS and other population-based cohorts, where a threshold of 0.25 has been shown to distinguish individuals with clinically meaningful frailty from those with more robust profiles [20].

#### Sociodemographic characteristics

We included the following variables: age in years and sex, marital status (married/civil union versus without a couple), and the number of completed years of formal education.

#### Social vulnerability index

This variable was created by combining various items suggestive of living conditions for everyone, including marital status, support provided by friends and family, social activities and hobbies, among others [21, 22]. The social vulnerability index (SVI) was created using the methods described [23] and has been previously used in MHAS [3]. A more detailed description can be found in Supplementary Table 3. To have clear estimates, the SVI was further categorized according to its mean/median (0.45) into two groups, for descriptive purposes it was left as a continuous variable.

#### Lifestyle

Smoking status was assessed based on the following questions: ‘Have you smoked more than 100 cigarettes or 5 packs in your lifetime; not including pipes or cigars?’ and ‘Do you smoke cigarettes now?’, resulting in three categories: never smoked, used to smoke and current smoking. Physical activity was defined as exercising ≥ 3 times a week in the previous year. Finally, risky alcohol use was defined as exercising ≥ 2 drinks per day for women and ≥ 3 drinks per day for men [24].

### Statistical analysis

We conducted a descriptive analysis of all the variables and bivariate analyses by survival status for baseline characteristics, using chi-square tests for all the variables (except for FI and SVI, where t tests were used). Descriptive statistics were used to report these findings.

In addition, Kaplan–Meier curves were plotted to assess the differences between performance status groups and other cancer-related variables; log-rank tests were used for statistical significance. Cox regression models were fitted to test the associations with mortality with hazard ratios (HR). Interaction terms between FI and performance status variables were included for the whole sample and were unadjusted and adjusted for baseline characteristics. All the statistical analyses were performed with the statistical software StataNow 18.5 (Stata Corp LLC, 4905 Lakeway Dr; College Station, TX, USA).

## Results

### Descriptive statistics

Our sample was composed of 318 individuals (Fig. 1), with a mean age of 68.02 years (SD 10.78), and most were women (62.57%). The main types of cancer found in our sample were breast cancer (26%), cervical cancer (24%) and endometrial cancer (23%) (see Fig. 1). Chemotherapy was the most common treatment (47.33%), surgery was part of the treatment for 33.96% of the sample, and radiotherapy was the least used reported treatment for 21.39% of the studied individuals (see Fig. 1).

Overall, 22.64% of the sample were physically active. With respect to smoking status, approximately one-third of the sample were former smokers (30.48%), and 9.09% were active smokers. Risky alcohol was present in 8.56% of the individuals. Statistically significant differences were observed for age, sex, and physical activity: deceased participants were older, more likely to be female, and less physically active compared to those who were alive at follow-up. Those who died had higher means for the frailty index, social vulnerability status, days in bed and performance status scores (see Table 1).

**Table 1:** Baseline characteristics of the study population stratified by survival status

	Deceased *n* = 123	Alive *n* = 195	*p* value
Age, mean (SD)	70.9 (0.92)	66.6 (0.67)	0.047
Female, n (%)	66 (66.9)	168 (53.7)	0.010
Physical activity, n (%)	12 (13.4)	60 (26.2)	0.015
Frailty index, mean (SD)	0.339 (0.153)	0.351 (0.011)	< 0.001
Social vulnerability index, mean (SD)	0.469 (0.106)	0.436 (0.109)	< 0.001
Days in bed, mean (SD)	23 (47.9)	7.3 (16.4)	< 0.001
Physical function score, mean (SD)	3.6 (3.02)	2.5 (2.53)	< 0.001
Smoking status, n (%) Never smoked Smoked in the past Currently smokes	69 (56.10) 39 (31.71) 15 (12.20)	157 (62.55) 75 (29.88) 19 (7.57)	0.272
Alcohol intake status, n (%)	9 (7.32)	23 (9.16)	0.549
Performance status categories, n (%) 0 1 2 3 4	1 (0.81) 3 (2.44) 26 (21.14) 32 (26.02) 61 (49.59)	11 (4.38) 20 (7.97) 71 (28.29) 89 (35.46) 60 (23.90)	< 0.001

FI: Frailty Index; SVI: Social Vulnerability Index; SD: Standard Deviation

Risky alcohol intake is defined as ≥ 2 drinks/day for women and ≥ 3 drinks/day for men

Performance status categories range from 0 (fully active) to 4 (bedridden > 50% of the time, with disability and no physical activity)

### Kaplan‒Meier survival estimates

**Fig. 2:**
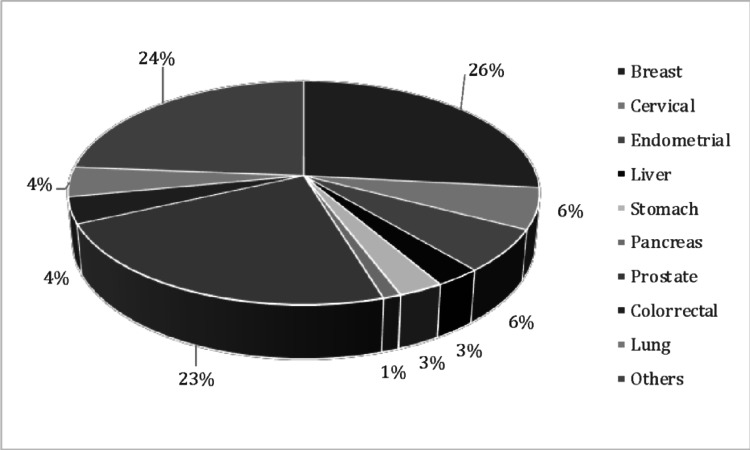
Frequency of the different types of cancer for the sample

The Kaplan–Meier curves further revealed greater survival in patients with better performance status (lower scores) – (0.81% mortality with performance status category 0) - which decreased with increasing scores (worse performance status). – (49.6% mortality with performance status category 4) (see Fig. 2). However, the group with higher mortality was the one categorized as 4, a statistically significant difference (*p* < 0.001).

### Cox regression models

Cox regression revealed that for low frailty levels, the social vulnerability index (HR 234.79; 95% CI 4.51–12222.8; *p* = 0.007) and performance status 2 (HR 5.46; 95% CI 1.24–24.04; *p* = 0.025) were significantly associated with increased risk of mortality, whereas age, sex, smoking status, and alcohol intake did not affect mortality risk. In contrast, among individuals with high frailty levels, age (HR 1.04; 95% CI 1.00–1.07; *p* = 0.039) was the only significant predictor of increased risk.

Additionally, when the performance status score was analyzed, a score of 4 clearly increased mortality 8-fold, but this association was lost in those with high frailty levels (see Table 2).

**Table 2:** Cox proportional hazards models for all-cause mortality stratified by frailty level

	Low frailty levels, HR (CI 95%, *p* value)	High frailty levels, HR (CI 95%, *p* value)
Age	1.02 (0.98–1.06, 0.373)	1.04 (1.00-1.07, 0.039)
Female	0.62 (0.28–1.36, 0.223)	0.56 (0.35–1.35, 0.281)
Social vulnerability index	1.91 (0.65–5.64, 0.237)	0.91 (0.44–1.87, 0.801)
Smoking status Never smoked Smoked in the past Currently smokes	Reference 1.21 (0.52–2.85, 0.664) 1.60 (0.41–6.28, 0.498)	Reference 0.95 (0.49–1.83, 0.864) 0.86 (0.33–2.35, 0.807)
Alcohol intake	0.66 (0.18–2.44, 0.538)	1.86 (0.51–6.81, 0.347)
Modified performance status	0 and 1 levels reference groups	
2	5.46 (1.24–24.04, 0.025)	0.52 (0.08–3.28, 0.490)
3	3.98 (0.84–18.73, 0.081)	1.15 (0.25–5.25, 0.855)
4	8.01 (1.50-42.66, 0.015)	3.13 (0.73–13.47, 0.126)

*HR: Hazard Ratio; CI: Confidence Interval.*

*Reference categories: For smoking*,* “Never smoked”; for Modified Performance Status*,* categories 0 and 1.*

*Low frailty = FI < 0.25; High frailty = FI ≥ 0.25.*

Social vulnerability index was categorized into < 0.45 and ≥ 0.45

## Discussion

**Fig. 3:**
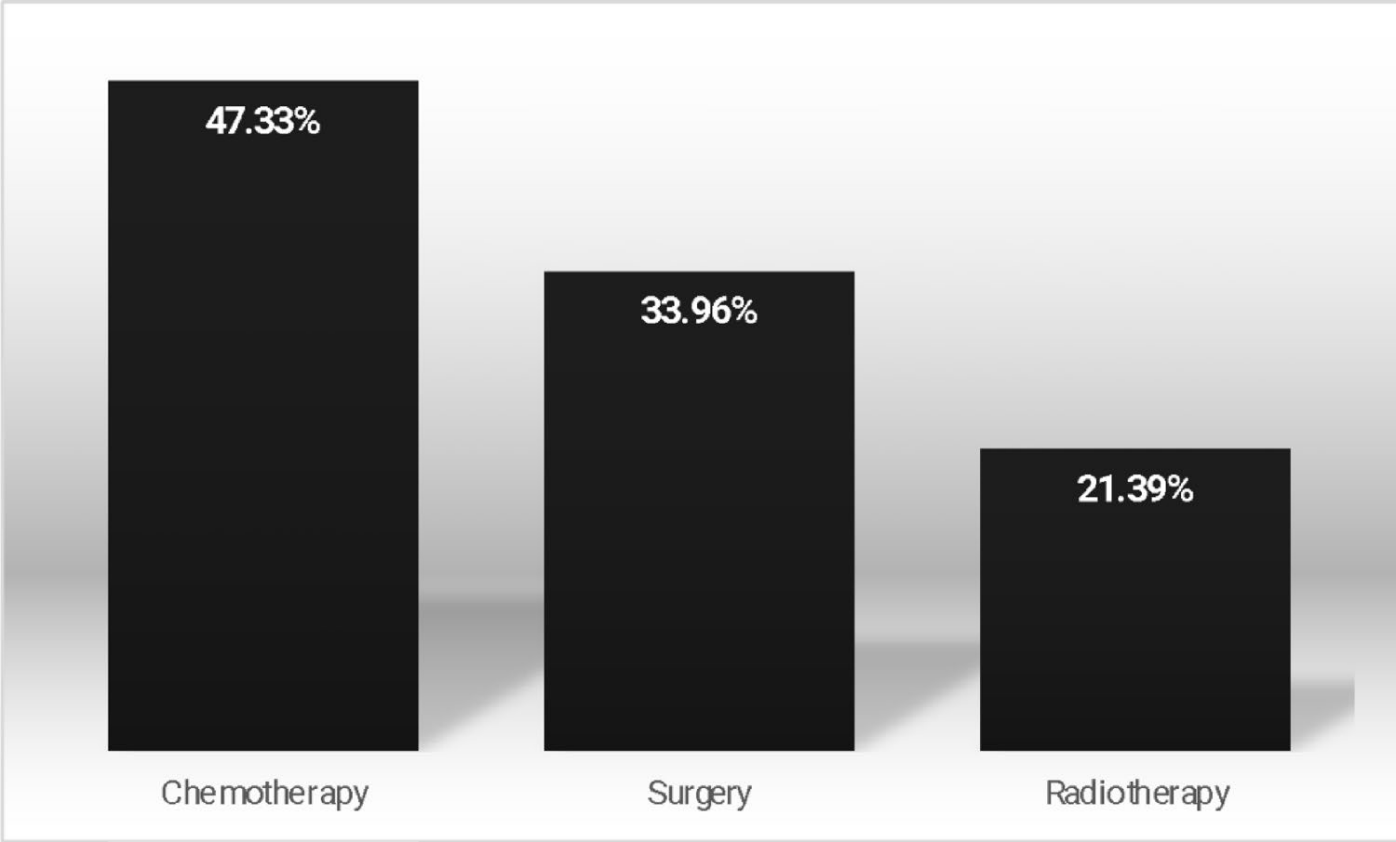
Frequency of the different types of treatment received as main therapy

**Fig. 4:**
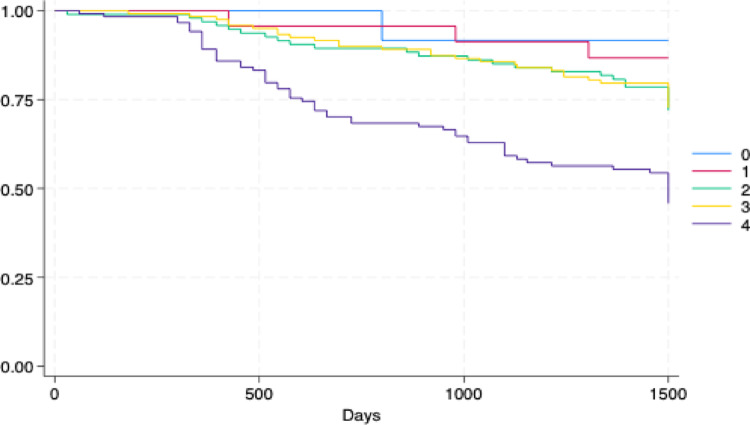
Kaplan–Meier survival curves by Modified Performance Status categories (0 to 4) showing cumulative probability of survival across follow-up time.*Higher performance status scores indicate worse physical functioning. p < 0.001 (log-rank test).

Figure 3 In older adults with cancer and low frailty, the composite performance status tool was associated with increased mortality risk. While the relationship was not strictly Linear, as category 3 did not show a statistically significant association, the effect was clear at modified performance status categories 2 and 4. In fact, previous reports have consistently demonstrated this association across different types of cancer [25–27]. However, our results showed that in individuals with high frailty levels, this composite tool was not associated with mortality. Interestingly, other variables such as the SVI also lost their significant association for mortality Fig.4

Older adults with cancer are at greater risk for mortality, treatment-related complications, hospitalizations and admissions to long-term care facilities [1]. Unlike younger patients, whose treatment decisions are typically based on outcomes such as survival and progression-free survival, for older adults, treatment goals should also include minimizing toxicity, maximizing quality of life and maintaining functional independence [28]. This broader focus has recently led to updates in guidelines from the American Society of Clinical Oncology (ASCO) and the Society of Geriatric Oncology (SGO), recommending the inclusion of comprehensive assessments of age-associated vulnerabilities in older patients receiving systemic cancer therapies [29–31].

Our findings are consistent with prior research showing that comprehensive geriatric assessment (CGA)-derived frailty scores offer greater prognostic value than performance status scales in older cancer patients. Numerous studies have shown that CGA not only predicts treatment-related toxicity and mortality more accurately but also provides a more nuanced assessment of age-related vulnerabilities [29–31]. For example, two randomized clinical trials published recently, the Geriatric Assessment for Patients 70 years and Older (GAP70+) and the Geriatric Assessment-Driven Intervention (GAIN), demonstrated the impact of an CGA on reducing the risk of toxicity and improving clinical outcomes in older adults. By identifying vulnerabilities (i.e., frailty) and tailoring treatment plans accordingly, these studies have shown that CGA can help mitigate treatment-related risks and improve overall patient well-being [2, 32].

However, while cost-effective and highly useful, the implementation of this assessment may be limited by resource and geriatricians’ availability across different settings [33]. Furthermore, our findings suggest that in older adults suffering from high levels of frailty, physical performance —typically a cornerstone of cancer prognosis—is less predictive of mortality than age as an isolated variable. This may be due to under recognition of frailty in settings where ECOG scores –or other similar tools– can be influenced by subjective assessments of a patient’s functional status. For example, it has been reported that older adults often receive worst ECOG scores despite having similar levels of physical activity as younger individuals [14]. This underestimation of functional status can lead to undertreatment. These results support growing calls for integrating frailty screening into oncologic decision-making, rather than relying solely on performance status measures. On the other hand, ECOG scores may be overestimated in older adults who are deemed “normal for their age,” potentially masking manageable conditions.

Moreover, recent studies suggest that clinicians have a limited ability to predict toxicity based on performance status alone, further emphasizing the need for additional evaluation for older adults suffering from cancer [34]. Our results also highlight the relevance of social vulnerability in predicting outcomes for older cancer patients, even when frailty is not present. On this matter, a study by Stuart et al. revealed that high social vulnerability scores were associated with patients presenting with advanced stages of cancer which lead to lower rates of surgery or chemotherapy, increasing the odds of dying due to these delays and incorrect decisions [35].

We recognize that our study has several Limitations. This is a secondary analysis of already available data; the latter implies that we created our variables based on information that had been collected in a standardized fashion but that were not collected specifically for our research question. Furthermore, different ways of assessing frailty exist; if a different approach is used, the results may differ. However, our study also has various strengths to consider. For example, this is a representative cohort of individuals followed for 20 years. This allows our conclusions to represent not only a large population but also a significant period, indicating that despite changes in available therapies, frailty continues to modulate the predictive ability of prognostic scores in older adults with cancer. Furthermore, lifestyle factors such as risky alcohol intake and smoking, as well as social vulnerability risk, which are known to be linked to worse outcomes, were considered covariates, which allows conclusions to be of greater strength. On the other hand, the final sample was reduced due to the selection criteria, and could have impacted the confidence intervals shown in the final regression. Finally, the tumors included in this work do not represent the current cancer landscape in Mexico, since we were limited to those available in MHAS questionnaires

## Conclusion

Our results highlight the importance of frailty as a key element in treatment decision-making and outcomes in older cancer patients. While performance status remains a valuable tool, its predictive value diminishes when frailty is present. The latter reflects the complexity of assessing prognosis in older adults with cancer, where factors such as frailty may overshadow other clinical markers, making a geriatric assessment a cornerstone of rational treatment in this group of patients.

## Data Availability

Data is provided within the manuscript or supplementary information files. The databases containing all information are available at mhasweb.org.
